# Recent Advances and Innovations in the Preparation and Purification of In Vitro-Transcribed-mRNA-Based Molecules

**DOI:** 10.3390/pharmaceutics15092182

**Published:** 2023-08-23

**Authors:** Jingjing Zhang, Yuheng Liu, Chao Li, Qin Xiao, Dandan Zhang, Yang Chen, Joseph Rosenecker, Xiaoyan Ding, Shan Guan

**Affiliations:** 1 National Engineering Research Center of Immunological Products, Third Military Medical University, Chongqing 400038, China; jjzhang2022@163.com (J.Z.); yhliu2022@163.com (Y.L.); lichao687@163.com (C.L.); xz953179866@163.com (Q.X.); zhang1995@163.com (D.Z.); y_chen1996@163.com (Y.C.); 2 Department of Pharmacology, College of Pharmacy, Chongqing Medical University, Chongqing 400016, China; 3 Department of Pediatrics, Ludwig-Maximilians University of Munich, 80337 Munich, Germany; joseph.rosenecker@med.uni-muenchen.de

**Keywords:** IVT mRNA, vaccine, purification strategy, sequence design, SARS-CoV-2

## Abstract

The coronavirus disease 2019 (COVID-19) pandemic poses a disruptive impact on public health and the global economy. Fortunately, the development of COVID-19 vaccines based on in vitro-transcribed messenger RNA (IVT mRNA) has been a breakthrough in medical history, benefiting billions of people with its high effectiveness, safety profile, and ease of large-scale production. This success is the result of decades of continuous RNA research, which has led to significant improvements in the stability and expression level of IVT mRNA through various approaches such as sequence optimization and improved preparation processes. IVT mRNA sequence optimization has been shown to have a positive effect on enhancing the mRNA expression level. The innovation of IVT mRNA purification technology is also indispensable, as the purity of IVT mRNA directly affects the success of downstream vaccine preparation processes and the potential for inducing unwanted side effects in therapeutic applications. Despite the progress made, challenges related to IVT mRNA sequence design and purification still require further attention to enhance the quality of IVT mRNA in the future. In this review, we discuss the latest innovative progress in IVT mRNA design and purification to further improve its clinical efficacy.

## 1. Introduction

Decades ago, IVT mRNA was developed, but its application was limited due to instability and high immunogenicity [1]. However, in recent years, the prevalence of COVID-19, caused by the Severe Acute Respiratory Syndrome Coronavirus 2 (SARS-CoV-2), has spurred the clinical translation of IVT mRNA to new heights. As a revolutionary innovation, mRNA vaccines have been developed at an unprecedented speed in the history of vaccine development. To date, the US Food and Drug Administration (FDA) has approved two mRNA vaccines [2,3,4]. These two mRNA vaccines have played a significant role in controlling the COVID-19 epidemic and have safely protected many recipients from SARS-CoV-2 [3,5,6]. The success of these approved mRNA vaccines has stimulated substantial interest in the application of the IVT mRNA technique [7]. This interest can be seen in the fact that numerous mRNA vaccines aiming to address infectious diseases other than COVID-19 are being tested in clinical trials (Table 1).

The mRNA vaccine platform offers several unique advantages over traditional vaccine approaches in addressing epidemic diseases [8]. For instance, compared to mRNA vaccines, inactivated vaccines and subunit vaccines (peptide or protein-based vaccines) generally share relatively lower immunogenicity, and could not induce potent humoral immunity and cellular immunity [9]. Unlike live attenuated vaccines, mRNA vaccines carry no risk of infectivity. Compared to DNA vaccines, there is no risk of integration into host DNA [10]. Moreover, mRNA does not need to cross the nuclear barrier, and the implication is mRNA does not need to access genomic DNA, allowing it to transfect slow-dividing or static cells. After protein expression, mRNA degrades quickly, ensuring controlled antigen exposure and minimizing the risk of tolerance induction [11,12]. mRNA molecules can easily enter the cytoplasm to translate target proteins, with high efficiency and speed. Furthermore, IVT mRNA production is rapid and scalable, and with a sufficient plasmid template, raw materials, and enzymes, the IVT mRNA required to produce a million mRNA vaccines can be obtained in a 5 L bioreactor [13]. Since IVT mRNA can be produced cell-free, there is a wide range of raw material options available, significantly reducing vaccine production costs [14]. This flexibility is particularly noteworthy, as the coding sequence of IVT mRNA can be designed for any type of protein, allowing for the rapid development and production of vaccines in response to pathogen mutation [15]. Thus, the mRNA vaccine platform holds immense value for the rapid iteration of vaccines.

Although IVT mRNA vaccines have many advantages, there are still several challenges that need to be addressed in order to further develop them, such as that (1) mRNA is unstable, leading to special conditions for mRNA vaccines, which need to be kept away from light at low temperatures. In the process of clinical transportation and storage, a lot of materials are needed [16]. (2) They are easily degraded with enzyme digestion in the body [7]. (3) They may induce a strong immune response when entering humans, causing inflammation and other discomfort, such as local irritation or an allergy [2]. (4) The bacterial target antigen will be glycosylated by the host cell, which may interfere with the generation of desired immune responses. It is necessary to note that factors intrinsic to mRNA itself can greatly affect the safety of mRNA vaccines and drug formulations. Fortunately, there have been many breakthroughs in recent years, including the optimization of mRNA preparation processes, sequence optimization, and various delivery technologies [17]. For example, mRNA is capped and tailed in various ways to improve its stability, and chemical modifications of nucleotides have been widely applied to reduce the immunogenicity of mRNA [18]. Improving the IVT mRNA purification process is also crucial, as impurities mixed within the crude IVT mRNA product can cause unwanted immunogenicity [19]. Researchers have been working hard to improve the IVT mRNA purification process, with chromatography being a highly selective technology that has been continuously explored [20]. At present, delivery systems for IVT mRNA vaccines are continuously being improved to enhance delivery efficiency [21,22,23,24]. This review will focus on the development and current progress of optimization strategies for the preparation and purification of IVT mRNA, as well as key issues encountered with current techniques.

## 2. Latest Optimization Strategies for IVT mRNA Sequence Design

**Figure 1:**
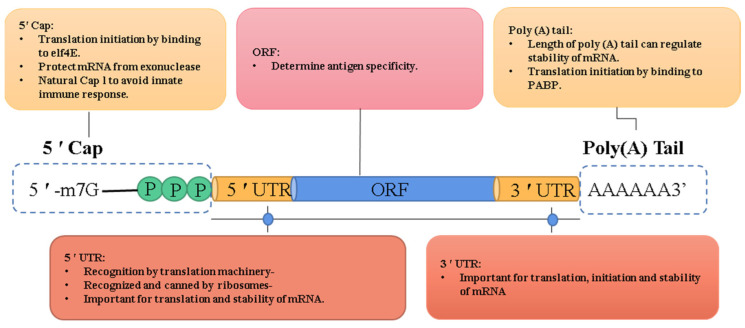
The five key domains of IVT mRNA and their function. IVT mRNA contains five structural elements: a 5′ cap containing 7-methylguanosine linked through a triphosphate bridge to a 2′-O-methylated nucleoside, flanking 5′ and 3′ UTRs, an ORF, and a poly(A) tail.

Normally, IVT mRNA is composed of five structural elements, including the 5′ cap, 5′ untranslated regions (UTRs), open reading frame (ORF), 3′ UTRs, and poly(A) tail (Figure 1). Each of them can serve as a modification site to improve the stability, immune response, and expression profile of the mRNA [25].

### 2.1. ORF

The ORF is particularly important because it contains the coding sequences that are crucial for the immunogenicity of the antigen. By replacing rare codons with more frequently occurring ones, the efficiency of translation can be improved while maintaining the protein sequence, because the abundant homologous tRNA in the cytoplasm can be reused near the ribosome [26]. Although ORF has higher plasticity than non-coding regions [27,28], it is worthy to note that the slow translation rate of rare codons is key for the formation of the tertiary structure of proteins, particularly for antigens with complex structures [29].

In addition to codon optimization, nucleosides in the mRNA sequence can also be modified to optimize translation levels. Chemical modifications, such as the use of pseudo-uridine and N1-methylpseudouridine [30], can prevent recognition by pattern recognition receptors (PRR) and reduce the risk of triggering an innate immune response [10]. The COVID-19 mRNA vaccines produced by Moderna and Pfizer BioNTech have incorporated modified nucleosides to ensure efficient antigen expression and minimize adverse immune effects [31]. While optimizing the ORF and nucleoside modifications are significant, other structural elements also play a role in the stability and expression of IVT mRNA. By modifying the length, type, and base composition of these elements, the half-life of IVT mRNA can be prolonged, an unnecessary immune response can be eliminated, and the expression level of IVT mRNA can be improved. Researchers continue to explore new optimization strategies for IVT mRNA preparation and purification techniques.

### 2.2. 5′ Cap

**Figure 2:**
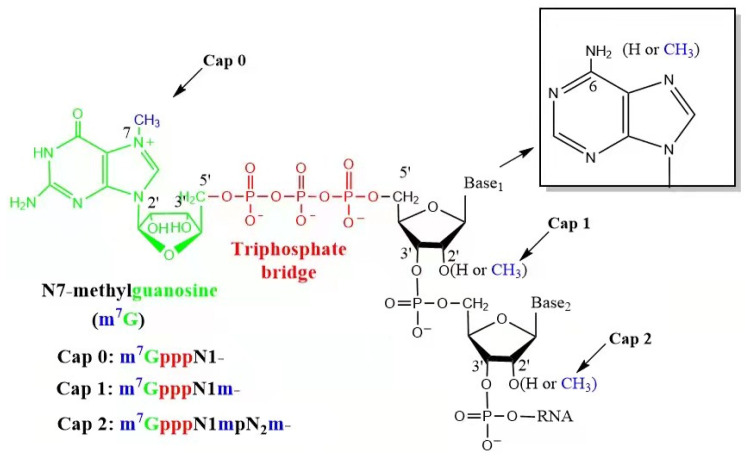
The types of an mRNA cap. IVT mRNA contains three types of caps, i.e., Cap 0, Cap 1, and Cap 2.

The 5′ cap (Figure 2) plays a crucial role in mRNA stability and translation efficiency by protecting mRNA from exonuclease degradation [32]. Even a slight modification of the 5′ cap, such as changing the characteristics and methylation status of the first nucleotide, can significantly affect the mRNA expression level in living cells. Eukaryotic mRNA has several cap structures, including Cap 0, Cap 1, and Cap 2. Cap 0 is the most basic structure, consisting of m7GpppNp. However, mRNA with a Cap 0 structure may be recognized by the host as exogenous RNA, and Cap 0 has affinity with the innate immune receptor retinoic-acid-induced gene I (RIG-I), whose activation triggers the Type I IFN response (IFN I) [33]. The cap structures of native endogenous mRNA are mainly Cap 1 or Cap 2, which have high translation efficiency. Cap 1 (m7GpppN1mp) is an improved structure, with a methylated 2′-OH on the first nucleotide [34], reducing the activation of PRR and improving translation efficiency [35]. Therefore, Cap 1 is commonly used for capping mRNA vaccines [36]. The mRNA Cap 2′-O-methyltransferase, which uses S-adenosylmethionine (SAM) as the methyl donor, is encoded by recombinant *E. coli*-expressed vaccinia virus DNA. It adds methyl groups at the 2′-O site of the first nucleotide next to the Cap 0 structure to form mRNA with a Cap 1 structure, enhancing mRNA translation efficiency and reducing immunogenicity. This enzyme specifically recognizes the 7-methylguanosine cap structure (m7Gppp, Cap 0) and will not act on RNA with pN, ppN, pppN, or GpppN at the 5′ end.

**Figure 3:**
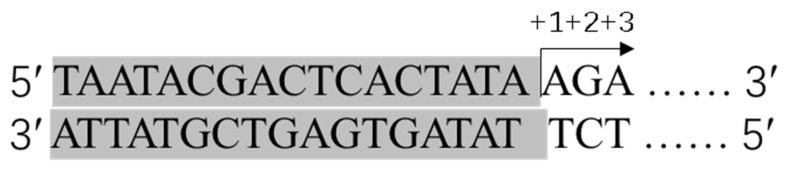
T7 RNA polymerase promoter sequence (grey) with initiation sequence required for CleanCap AG. Arrow indicates transcription start site with nucleotide positions shown above.

Currently, two methods have been developed for capping IVT mRNA: enzymatic capping and co-transcriptional capping using cap analogues (m7G-ppp-X). The enzymatic capping reaction can synthesize caps at the 5′ end of mRNA with a 100% capping efficiency using two enzymes. Vaccinia capping enzyme (VCE) can cap the mRNA to generate a Cap 0 structure, which can then be methylated to a Cap 1 structure using 2′-O-methyltransferase (2′O-MTase) [37]. Moderna has successfully obtained the Cap 1 structure of mRNA-1273 through enzymatic capping [15]. In contrast, co-transcriptional capping using cap analogues does not require a second enzymatic reaction and only needs a nucleic acid precipitation or purification process in the IVT process [37]. Therefore, co-transcriptional capping can be a more efficient process, presumably leading to a lower cost of time and goods in a research laboratory [38,39], but the first-generation cap analogue resulted in Cap 0 structures with low capping efficiency (60–80%) [40]. The technology for a one-pot synthesis of capped IVT mRNA is improving with the development of new cap analogues. A novel co-transcriptional capping method called CleanCap has been found to generate a natural Cap 1 structure for Cas9 mRNA, which is commonly used for genome editing [30]. This method has a yield of IVT mRNA with a 94% (or higher)-Cap 1 structure by using Clean Cap^®^ Reagent AG; it is worth noting that when using CleanCap for co-transcriptional capping, the addition of bases A and G at positions +1 and +2 is required, respectively, in the T7 promoter. As the CleanCap trimer binds to the +1 and +2 nucleotides of the template through complementary base pairing, this is followed by the incorporation of the complementary NTP at the +3 position (Figure 3) [41]. Recently, a study developed hydrophobic photocaged tag-modified cap analogues, which separate capped mRNA from uncapped mRNA with reversed-phase high-performance liquid chromatography. Subsequent photoirradiation recovers footprint-free native capped mRNA. In this work, the new approach provides 100% capping efficiency with versatility applicable to 650 nt and 4247 nt mRNA [42]. When choosing a capping scheme, the cost of production and the impact of capping analogues on production costs should be considered (Table 2) [43]. Furthermore, to optimize mRNA translation, uncapped IVT mRNA should be treated with phosphatases to avoid recognition by the innate immune system, as RIG-I (a receptor that recognizes abnormal viral mRNA, recognizing the 5′ triphosphate of uncapped mRNA) can lead to abolished mRNA translation, so both methods of adding caps require this step to be taken into consideration because the actual capping efficiency is not always 100% [44].

### 2.3. Poly(A) Tail

**Figure 4:**
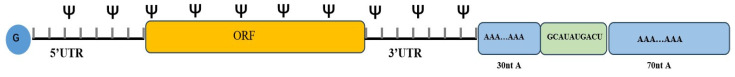
An exemplary structure of segmented poly(A). This poly(A) tail is applied in BioNTech’s COVID-19 mRNA vaccine.

The poly(A) tail is another critical component of IVT mRNA [45,46]. It binds to multiple poly(A)-binding proteins (PABPs) [47], which recruit eukaryotic initiation factor 4G (eIF4G) and enhance the affinity between the cap and poly(A) tail. This interaction forms an mRNA loop that prevents mRNA degradation and promotes ribosome re-entry for translation [48]. Consequently, the poly(A) tail indirectly regulates translation efficiency. Typically, actively translated mRNAs in mammalian cells possess 100–250 adenosine residues [33,49]. Studies have demonstrated that optimizing the length of the poly(A) tail improves translation efficiency and mRNA stability [48,49]. In IVT, the generally accepted length for the prevailing view is between 110 and 160 nt, and the highest expression is achieved when the length of the poly(A) tail reaches 120 nt [50]. Recent studies have revealed that mRNAs with poly(A) tails longer than 300 nt also exhibit decent translation efficiency [51]. Additionally, many highly expressed genes in eukaryotes possess short poly(A) tails, which appear to be tailored to form a closed loop structure [52]. In summary, different lengths of poly(A) tails need to be optimized for different mRNA to achieve optimal mRNA function. There are two main approaches to adding a poly(A) tail to IVT mRNA. The first approach involves the traditional enzymatic reaction, where the poly(A) tail is added to the 3′ end of mRNA. But accurately controlling the length of the poly(A) tail using this method, particularly for long IVT mRNA, can be challenging, which may affect quality assurance [53]. The second approach involves designing the DNA template to include a poly(A) sequence, which is then transcribed in vitro along with the target mRNA to generate a poly(A) tail. This approach allows for adjusting the length of the poly(A) tail through template design, eliminating variability caused by enzymatic polyadenylation with poly(A) polymerase [46]. When a long tail (more than 100 nt) is required, plasmid-DNA-encoded poly(A) will possibly recombine during bacterial amplification [54]. Previous studies reported the generation of spontaneous deletion mutants during the amplification of plasmids starting with ~100 bp of poly (dA:dT) sequences [55]. For longer poly(A)s (more than 150 nt), the instability is too high to allow the isolation of any single positive clone [51]. Using the segmented poly(A) method could significantly reduce plasmid recombination in *E. coli* without any negative effects on mRNA half-life and protein expression [54]. Furthermore, subsequent studies have successfully improved the stability of IVT mRNA by adding a short UGC linker to the poly(A) tail [56]. This strategy was employed by BioNTech in developing the COVID-19 mRNA vaccine, where a 10-nt UGC linker (A30LA70) was inserted between poly(A) sequences (Figure 4) [57]. Therefore, to optimize the length and stability of the poly(A) tail, specific optimization strategies, such as segmenting the tail by adding a UGC linker, should be considered.

### 2.4. UTR

UTRs are other regulatory elements located on both sides of the ORF (open reading frame) of mRNA. The 5′ and 3′ UTRs play distinct roles in regulating translation and maintaining the stability of IVT mRNA by interacting with RNA-binding proteins [58]. The 5′ UTR contains the binding site of the translation complex, thereby controlling the translation efficiency of the downstream ORF [59,60]. On the other hand, the 3′ UTR typically contains mRNA degradation signals, including AU-rich sequences that aid in poly(A) tail removal during mRNA degradation [61,62,63]. By replacing the AU-rich sequences of an unstable mRNA with sequences from a more stable counterpart, the half-life of the mRNA can be prolonged [64]. To achieve higher expression and stability [65], several methods are currently employed. These methods include selecting natural UTRs from highly expressed genes (e.g., α- and β-globin) for IVT mRNA synthesis. Additionally, a screening method has been reported to identify the optimal combination of 5′ and 3′ UTRs that enhance therapeutic mRNA expression levels [66]. Apart from screening naturally occurring UTRs, artificially constructed UTRs are designed to be optimized for specific target cells and clinical applications. These engineered UTRs minimize mRNA degradation by excluding miRNA-binding sites and AU-rich regions in the 3′ UTR [67,68]. Furthermore, they minimize regions that prevent ribosomes from scanning the mRNA transcript, such as sequences with secondary and tertiary structures (e.g., hairpins) in the 5′ UTR [60]. More recently, bioinformatics or deep learning technology has been introduced to design new UTRs and predict mRNA translation efficiency [69].

## 3. IVT mRNA Purification

### 3.1. The Importance of IVT mRNA Purifications

**Figure 5:**
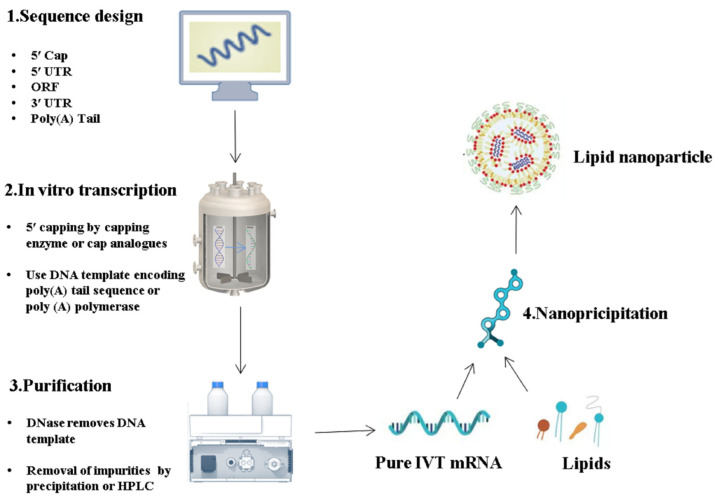
Schematic illustration on the key production process of the IVT mRNA and its lipid-based formulation. (1) Once the genome of a pathogen has been revealed, a sequence for the target antigen is designed and inserted into a plasmid DNA construct. (2) Plasmid DNA is transcribed into mRNA by bacteriophage polymerases in vitro and (3) mRNA transcripts are purified with high-performance liquid chromatography (HPLC) to remove contaminants and reactants. (4) Purified mRNA is mixed with lipids in a microfluidic mixer to form lipid nanoparticles.

The synthesis of high-quality IVT mRNA is crucial for the success of subsequent research, as it directly affects downstream vaccine preparation processes and the efficacy of mRNA vaccines. The production process of mRNA vaccines involves several steps, including target antigen sequence design, DNA template preparation, IVT mRNA, mRNA purification, and LNP formulation (Figure 5).

During the in vitro synthesis of mRNA, various components, including a DNA plasmid, RNA polymerase, metal ion coenzyme factors, and nucleotide starting materials, may inadvertently mix in the final product. If the plasmid remains intact and penetrates the cell plasma after administration, it could potentially lead to genome integration. Moreover, plasmids produced through microbial fermentation may contain impurities such as endotoxin and proteins, which have high immunogenicity and can cause inflammation if not removed through chromatographic separation prior to in vitro transcription. Additionally, the enzymes involved in the in vitro transcription may introduce pollutants and exogenous factors [70], which, if not removed, could induce pro-inflammatory cytokines and inflammation. The unpurified IVT mRNA product may also contain unwanted RNA molecules, including truncated or abnormal transcription, uncapped mRNA, and double-stranded RNAs (dsRNAs), which can negatively impact the function of IVT mRNA. The elimination of dsRNA from IVT mRNA is crucial to enhance mRNA translation and minimize the induction of cytokines and unwanted inflammation responses [19]. Furthermore, nucleoside triphosphate substrates (NTPs) may persist in mRNA transcripts, potentially activating the neuroinflammatory mechanism in the central nervous system [71].

The efficient removal of the aforementioned impurities is essential to improve mRNA translation levels and prevent the activation of undesirable immune responses, thereby obtaining non-immunogenic IVT mRNA with enhanced translation efficiency. Failure to effectively control these impurities can result in strong rejection reactions in patients during the final clinical application, posing a threat to their lives. Therefore, the development of efficient methods for the purification of IVT mRNA is needed [72,73,74]. In fact, the purification step is considered as the most challenging aspect in the large-scale production of IVT mRNA [75]. To ensure the purity and safety of IVT mRNA, rigorous purification processes must be implemented to eliminate unwanted components and byproducts. Although the specific purification process for BNT162b2 and mRNA-1273 have not been disclosed, the last advancements in mRNA technology and the growing demand for purification have led to the exploration of the several commonly used purification methods in mRNA preparation (Table 3), which are briefly discussed in the following sections [76].

### 3.2. Precipitation Methods

The conventional method for purifying mRNA in a laboratory setting is relatively simple. It involves DNA enzyme digestion to eliminate the DNA template, followed by mRNA precipitation using alcohol or isopropanol [77], and monovalent cations like sodium or ammonium ions [78]. However, the use of ammonium or sodium acetate in this method can lead to a high-concentration salt solution precipitate, which requires additional desalination techniques for removal. Another commonly employed method of mRNA separation is lithium chloride (LiCl) precipitation [10,32], which has the advantages of not precipitating DNA, protein, or carbohydrates, and is easily washed out due to its high solubility in an ethanol solution. These precipitation methods do not effectively remove abnormal mRNA, including truncated RNA fragments and dsRNAs, which can adversely impact mRNA function. Moreover, if the washing step is not performed thoroughly, cationic impurities may persist and pose potential safety hazards.

### 3.3. Chromatography Purification Methods

Similar to the development of recombinant protein purification, the field of mRNA research is also moving towards chromatographic methods. Among these methods, HPLC is considered the gold standard for mRNA purification in laboratory settings [19]. In recent years, several strategies using chromatography purification technology have been explored to overcome the increasing challenges in IVT mRNA purification.

Size-exclusion HPLC (SEC) is a chromatographic technique that separates molecules based on their size, making it the simplest form of chromatography for purifying oligonucleotides [79]. The first published protocol for the large-scale synthesis and purification of RNA oligonucleotides was achieved using SEC. By combining SEC with fast protein liquid chromatography (FPLC) [80], researchers can effectively remove unreacted nucleotides, enzymes, short transcripts, and high-molecular-weight DNA templates from the desired IVT mRNA products [81,82]. FPLC is a modern liquid chromatography similar to HPLC in principle. It is a delightful innovation of HPLC in recent years. This combination (SEC-FPLC) is performed under non-denaturing conditions, avoiding precipitation steps that may lead to mRNA degradation and a low recovery rate [83]. Moreover, this method can easily be scaled up for large-scale purification, resulting in high yields of pure mRNA products. The SEC-based method still requires several time-consuming steps such as protein removal via phenol/chloroform extraction, desalination, and concentration [19]. Additionally, removing impurities of a similar size, such as dsDNA, can be challenging.

**Figure 6:**
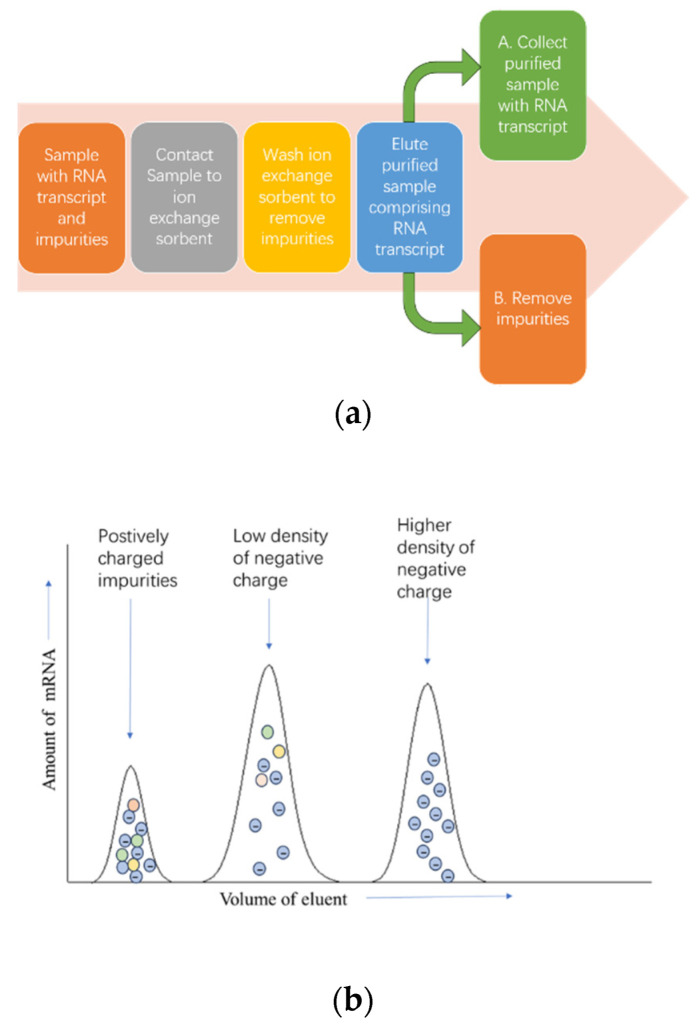
The separation process of IEC. (**a**) The separation process of crude samples from loading to obtaining purified samples; (**b**) the peak order of different substances in IEC.

Ion exchange HPLC (IEC) is another effective method for the large-scale purification of IVT mRNA. Given the polyanionic nature of mRNA molecules, ion exchange matrices have been extensively explored for chromatographic separation according to the charge difference between the target mRNA species and the different impurities [84]. The crude transcription reaction is applied directly to weak anion-exchange chromatography, and T7 RNA polymerase and unincorporated NTPs, which do not bind to the column matrix, are found in the flow-through. Small abortive transcripts, the desired RNA product, and the plasmid DNA template are separated on the column over a shallow salt gradient (Figure 6a,b) [85]. Since IEC separation is carried out under aqueous conditions without using expensive eluents, it is scalable and cost-effective. IEC chromatography is commonly used for oligonucleotide purification in medium- to large-scale manufacturing processes [70].

Reversed-phase HPLC (RP-HPLC) is a commonly used approach to remove double-stranded RNA (dsRNA), which is a major impurity in IVT mRNA resulting from the characteristics of RNA polymerases [86]. There are many types of dsRNA sensors in the cytoplasm, such as RIG-I, melanoma differentiation-associated gene 5 (MDA-5), dsRNA-dependent kinase (PKR), and oligoadenylate synthetase (OAS) [87]; they trigger the innate immune signal pathway by stimulating RIG-I and MDA-5 [88]. The removal of dsRNA can not only reduce the non-specific immunogenicity of mRNA but also improve the expression efficiency of mRNA [10]. The reversed-phase HPLC (RP-HPLC)-based method is a commonly used approach to remove dsRNA [89]. In RP-HPLC, the negatively charged sugar–phosphate backbone of IVT mRNA pairs with quaternary ammonium compounds in the mobile phase to make them lipophilic, allowing them to interact with the stationary phase of the reverse-phase chromatographic column; meanwhile, dsRNA could be eluded down early along with the mobile phase. After elution with an appropriate solvent (such as acetonitrile), the target mRNA could be obtained while maintaining a high recovery [90]. RP-HPLC also has some challenges and drawbacks, such as the toxicity of the organic solvent used in the elution phase and the need for the further purification of the recovered mRNA product to meet therapeutic standards [75]. RP-HPLC columns are typically placed in an incubator that keeps the temperature at 75 °C to avoid self-complementary or the aggregation of IVT mRNA with GC-rich sequences and to improve resolution [91], but this may not be conducive to maintaining the stability and biological activity of the target mRNA. Additionally, the loading capacity of RP-HPLC columns is limited, and the process can be subject to high temperatures and external forces. As a result, safer and more cost-effective purification methods have been developed and applied in recent research.

Affinity chromatography has been used for the purification of mRNA since the 1970s [92]. Currently, the most widely used and successful method involves oligo-deoxythymidine acid (oligo-dT). The single-strand sequence of oligo-dT is typically utilized to capture mRNA in laboratory applications by binding to the poly(A) tail in mRNA. Polyadenylated mRNA forms a stable hybrid with oligo-dT under high-salt conditions. The hybrid can be destabilized by removing the salt, thereby releasing the mRNA; this process will retain and then simultaneously elute all species with poly-A tails, while the impurities such as DNA templates and dsRNA can be effectively removed [93,94,95]. The affinity-based chromatographic isolation of mRNA is a robust and straightforward technique that serves as an industrial platform, producing high-purity products suitable for current good manufacturing practice (cGMP). The technique has some drawbacks. For instance, its binding capacity is limited by mRNA length and the loading concentration of salt in the loading phase, and it is a less cost-effective process compared to the traditional precipitation method because of the pricey fillers [89].

Cellulose chromatography is a relatively new alternative to HPLC for removing dsRNA from IVT mRNA [75]. This method utilizes the ability of dsRNA to bind with cellulose in the presence of ethanol. After purification, the mRNA recovery rate exceeds 65%, with a dsRNA removal rate of over 90% [75]. The degree of binding between cellulose and dsRNA depends on the ethanol concentration, and it has been shown that an optimal choice for IVT mRNA purification on a cellulose column is 16% ethanol [75]. Although cellulose purification has been developed for the large-scale production of IVT mRNA, it remains unclear whether this method can distinguish between the inherent secondary structures of dsRNA and mRNA [86]. Currently, this purification method has been successfully applied to the purification of self-amplifying mRNA (saRNA) for the Zika virus, and its effectiveness has been confirmed with an enhancement in the efficacy of the saRNA vaccine [96].

### 3.4. Non-Chromatography Purification Method

In addition to chromatographic technologies, researchers are constantly searching for new and more cost-effective methods for purifying IVT mRNA. One such method is the use of specific RNase III enzymes to digest dsRNA and generate pure ssRNA. When transfected into T cells, it can significantly improve the effectiveness of killing tumours both in vivo and in vitro [97]. While this method has proven effective in removing dsRNA, there is a risk of damaging the mRNA’s secondary structure and increasing the cost of purification [98].

Another alternative is optimizing the IVT process itself. High-temperature IVT combined with template-encoded poly(A) tailing can synthesize high-purity IVT mRNA without requiring additional dsRNA purification [86]. Additionally, lowering the concentration of Mg^2+^ during the IVT process has been shown to reduce dsRNA generation [88], although this can also impact overall yield [86]. A recently reported method involves adding a dispersant to the transcription system to reduce the generation of dsRNA impurities. The amount of dispersant can then be adjusted to accurately control the content of dsRNA [98]. It is worth noting that dsRNA can have adjuvant properties that may be helpful for eliciting an immune response [13,37], and adjusting the amount of dsRNA can help achieve an appropriate balance of innate and adaptive immune responses.

Although efforts have been made to obtain high-purity IVT mRNA, the majority of the approaches are not cost-effective, which may limit their ability to meet the demand of the actual market. Tangential flow filtration (TFF) has emerged as a fast and efficient method for filtering and concentrating solutions containing biological molecules [99]. TFF refers to a filtration form where the direction of liquid flow is perpendicular to the direction of filtration. The traditional filtration method is mostly vertical filtration, and the flow direction of the liquid is consistent with the filtration direction. With the filtration process, the thickness of the filter cake layer or gel layer formed on the surface of the filter membrane gradually increases, and the flow rate gradually decreases. Therefore, vertical filtration can only handle small volumes of feed liquid. When applying TFF, the feed containing the biomolecular solution can flow tangentially and continuously through the filter surface of the TFF device, while the residual solution returns to the feed tank for recycling. Therefore, TFF technology can be used for the large-scale production of IVT mRNA [99], and sometimes can be combined with mRNA precipitation. Currently, the TFF method can be used to replace mRNA precipitation methods in many cases, including those in the production process of approved COVID-19 mRNA vaccines [15,100,101].

**Table 3:** Comparison of different purification methods for mRNA.

Methods		Advantages	Disadvantages
Precipitation method	Precipitation method	easy to operate	form large particles; abnormal mRNA; cationic impurities
Non-chromatography purification method	RNase III	effectively remove dsRNA	harm for the secondary structure of mRNA; increases the cost of purification process
	Lower concentration of Mg^2+^	reduce the dsRNA generation	affects the overall yield of the IVT process
	Add dispersant into the transcription system	controls the content of dsRNA	/
	TFF	fast and efficient	/
Chromatography	SEC	simple	removes unreacted nucleotides, enzymes, short abortion transcripts, and high-molecular-weight DNA templates; time-consuming; difficult to remove impurities of similar size
	IEC	scalable and cost-effective	/
	RP-HPLC	effectively removes dsRNA	toxic organic solvents; may not be conducive to maintaining the stability and biological activity of the target mRNA; loading capacity of column is limited
	Affinity HPLC	simple and reliable	low binding capacities and a less cost-effective process
	Cellulose chromatography	for large-scale production of IVT mRNA	unclear whether this method can distinguish the inherent secondary structure of dsRNA and mRNA

## 4. Conclusions

IVT mRNA holds great promise as a safer and faster alternative to established vaccines. Achieving optimal IVT mRNA involves efficient capping, the dephosphorylation of uncapped transcripts, the elimination of dsRNA, and the use of modified nucleosides, in addition to inherent properties such as poly(A) tails, 3′ and 5′ UTRs, and codon-optimized sequences. Understanding these mechanisms underlying the several factors will be crucial for further optimizing IVT mRNA. For instance, recent studies have revealed that the poly(A) tail of human mRNA can contain non-adenosine residues, with cytidine substitution near the tail end showing potential to enhance protein expression and prolong intracellular half-life [102]. However, there is still limited research on the underlying mechanisms.

Purifying IVT mRNA from the reaction mixture to meet clinical purity standards remains a costly process due to imperfect downstream processing. Additionally, the inherent instability of mRNA molecules necessitates extreme storage and transportation conditions, posing challenges for widespread application and distribution [103]. Enhancing the stability of mRNA is therefore a key area for improvement. Chromatography has shown to yield more stable mRNA compared to precipitation methods, eliminating factors that negatively impact mRNA quality [93].

Future advancements in IVT mRNA technology offer solutions to address the production cost and storage challenges. Ongoing improvements in mRNA design and purification techniques are expected to enhance storage and transportation convenience, making mRNA technology more accessible, particularly for economically disadvantaged countries. These advancements have the potential to expand the menu of therapeutic options of mRNA technology, including cancer immunotherapy [84], protein replacement therapy [65], and beyond [104]. The promising future of IVT mRNA technology holds the potential to revolutionize the healthcare industry. 

## Figures and Tables

**Table 1:** Clinical trials of mRNA vaccines for infectious diseases other than COVID-19.

Identifier	Target	Sponsor	Name	Route of Administration	Status	Phase
NCT05217641	HIV (Human Immunodeficiency Virus)	National Institute of Allergy and Infectious Diseases National Institutes of Health Department of Health and Human Services	BG505 MD39.3 BG505 MD39.3 gp151 BG505 MD39.3 gp151 CD4KO	I.M	Active, not recruiting	Ⅰ
NCT05398796	Nipah Virus	National Institute of Allergy and Infectious Diseases Moderna TX, Inc. (Cambridge, MA 02139, USA). National Institutes of Health Clinical Center	mRNA-1215	I.M	Recruiting	Ⅰ
NCT05430958	Coronavirus	Inovio Pharmaceuticals	INO-4800 INO-9112	I.M	Withdrawn	Ⅰ
NCT05414786	HIV-1	International AIDS Vaccine Initiative AURUM Tembisa Clinical Research Center for Family Health Research	mRNA-1644	I.P	Active, not recruiting	Ⅰ
NCT05127434	Respiratory Syncytial Virus	Moderna TX, Inc.	mRNA-1345	I.M	Recruiting	Ⅱ/Ⅲ
NCT03713086	Rabies	CureVac	CV7202	I.M	Completed	Ⅰ
NCT05624606	Influenza Immunization	Sanofi Pasteur	MRT5410	I.M	Not yet recruiting	Ⅰ/Ⅱ
NCT05553301	Influenza Immunization	Sanofi Pasteur	MRT5407	I.M	Recruiting	Ⅰ/Ⅱ
NCT05105048	Cytomegalovirus	Moderna TX, Inc.	mRNA-1647	I.M	Recruiting	Ⅰ
NCT05085366	Cytomegalovirus	Moderna TX, Inc.	mRNA-1647	I.M	Recruiting	Ⅲ
NCT04232280	Cytomegalovirus	Moderna TX, Inc.	mRNA-1647	I.M	Active, not recruiting	Ⅱ
NCT03382405	Cytomegalovirus	Moderna TX, Inc.	mRNA-1647/ mRNA-1443	I.M	Completed	Ⅰ
NCT05164094	Epstein–Barr Virus	Moderna TX, Inc.	mRNA-1189	I.M	Recruiting	Ⅰ
NCT03392389	Human Metapneumovirus and Human Parainfluenza	Moderna TX, Inc.	mRNA-1653	I.M	Completed	Ⅰ
NCT05581641	Malaria	BioNTech SE	BNT165b1	I.M	Not yet recruiting	Ⅰ
NCT04917861	Zika Virus	Moderna TX, Inc.	mRNA-1893	I.M	Active, not recruiting	Ⅱ
NCT04064905	Zika Virus	Moderna TX, Inc. Biomedical Advanced Research and Development Authority	mRNA-1893	I.M	Completed	Ⅰ
NCT03014089	Zika Virus	Moderna TX, Inc. Biomedical Advanced Research and Development Authority	mRNA-1325	I.M	Completed	Ⅰ
NCT05566639	Seasonal Influenza	Moderna TX, Inc.	mRNA-1010	I.M	Recruiting	Ⅲ
NCT05537038	Tuberculosis	BioNTech SE	BNT164a1/BNT164b1	I.M	Not yet recruiting	Ⅰ
NCT02888756	HIV	Rob Gruters Institut d’Investigacions Biomèdiques August Pi i Sunyer IrsiCaixa	iHIVARNA-01 Tri Mix	I.M	Terminated Has Results	Ⅱ
NCT05547464	Tuberculosis	BioNTech SE	BNT164a1/BNT614b1	I.M	Not yet recruiting	Ⅰ
NCT05415462	Seasonal Influenza	Moderna TX, Inc.	mRNA-1010	I.M	Active, not recruiting	Ⅲ
NCT04956575	Seasonal Influenza	Moderna TX, Inc.	mRNA-1010	I.M	Completed	Ⅰ/Ⅱ
NCT05333289	Seasonal Influenza	Moderna TX, Inc.	mRNA-1030/mRNA-102/mRNA-1010	I.M	Active, not recruiting	Ⅰ/Ⅱ
NCT02241135	Rabies	CureVac	CV7201	I.M	Completed	Ⅰ
NCT05606965	Influenza	Moderna TX, Inc.	mRNA-1010	I.M	Recruiting	Ⅱ
NCT05252338	Seasonal Influenza	CureVac GlaxoSmithKline	CVSQIV	I.M	Recruiting	Ⅰ
NCT03345043	Influenza A(H7N9)	Moderna TX, Inc.	mRNA-1851	I.M	Completed	Ⅰ
NCT03076385	Influenza A(H10N8)	Moderna TX, Inc.	mRNA-1440	I.M	Completed	Ⅱ
NCT05220975	RSV	Moderna TX, Inc.	mRNA-1345	I.M	Recruiting	Ⅲ
NCT04144348	hMPV/PIV3	Moderna TX, Inc.	mRNA-1653	I.M	Recruiting	Ⅲ
NCT04062669	Rabies	GlaxoSmithKline	GSK3903133A	I.M	Active, not recruiting	Ⅰ

The data were collected on the website https://www.clinicaltrials.gov (accessed on 3 December 2022). Clinical trials are regularly updated, and the locations and the number of participants are subject to change. HIV: Human Immunodeficiency Virus; I.M: Intramuscular Injection; I.P: Intraperitoneal Injection.

**Table 2:** The connections and differences between Enzymatic capping and CleanCap in IVT-mRNA synthesis.

	Enzymatic Capping	CleanCap
Enzymes	RNA polymerase; Capping enzyme	RNA polymerase
Reaction steps	Transcription and Capping	a one-pot synthesis
Purification steps	2	1
Other materials	/	a cap analogue
T7 promoter	TAATACGACTCACTATAGGG	TAATACGACTCACTATAAGA
Same materials	DNA template; Magnesium-containing buffer; RNase inhibitor, ATP/GTP/CTP/UTP; Inorganic pyrophosphatase	

## Data Availability

The data presented in this study are available on request from the corresponding author. The data are not publicly available due to privacy restrictions.
